# Attention-based neural networks for clinical prediction modelling on electronic health records

**DOI:** 10.1186/s12874-023-02112-2

**Published:** 2023-12-07

**Authors:** Egill A. Fridgeirsson, David Sontag, Peter Rijnbeek

**Affiliations:** 1 Department of Medical Informatics, Erasmus University Medical Center, Doctor Molewaterplein 40, 3015 GD Rotterdam, the Netherlands; 2 Institute for Medical Engineering & Science, Massachusetts Institute of Technology, Cambridge, MA USA

**Keywords:** Clinical prediction models, Deep learning, Electronic health records

## Abstract

**Background:**

Deep learning models have had a lot of success in various fields. However, on structured data they have struggled. Here we apply four state-of-the-art supervised deep learning models using the attention mechanism and compare against logistic regression and XGBoost using discrimination, calibration and clinical utility.

**Methods:**

We develop the models using a general practitioners database. We implement a recurrent neural network, a transformer with and without reverse distillation and a graph neural network. We measure discrimination using the area under the receiver operating characteristic curve (AUC) and the area under the precision recall curve (AUPRC). We assess smooth calibration using restricted cubic splines and clinical utility with decision curve analysis.

**Results:**

Our results show that deep learning approaches can improve discrimination up to 2.5% points AUC and 7.4% points AUPRC. However, on average the baselines are competitive. Most models are similarly calibrated as the baselines except for the graph neural network. The transformer using reverse distillation shows the best performance in clinical utility on two out of three prediction problems over most of the prediction thresholds.

**Conclusion:**

In this study, we evaluated various approaches in supervised learning using neural networks and attention. Here we do a rigorous comparison, not only looking at discrimination but also calibration and clinical utility. There is value in using deep learning models on electronic health record data since it can improve discrimination and clinical utility while providing good calibration. However, good baseline methods are still competitive.

**Supplementary Information:**

The online version contains supplementary material available at 10.1186/s12874-023-02112-2.

## Introduction

With the digital medicine revolution and personalized medicine more clinical prediction models are being built to aid in decision making in healthcare [1, 2]. With more of healthcare systems utilizing electronic health record (EHR) systems the potential availability of big databases to develop those models is increasing. However, with all this data there are challenges. Data is stored in different formats and different terminology systems. This has been addressed through the Observational Medical Outcomes Partnership Common Data Model (OMOP-CDM) maintained by the Observational Health Data Sciences and Informatics (OHDSI) community [3]. Additionally, solutions like the Fast Healthcare Interoperability Resources (FHIR) standard provide a complementary approach to enhancing data exchange and interoperability in healthcare [4]. The OMOP-CDM has enabled the use of common methods and tools across a large network of databases. This includes tools for predictive modeling such as the patient-level prediction (PLP) framework [5].

For predictive modelling the field of deep learning has had a heavy impact on the world in the last decade [6, 7]. In deep learning a layered approach is used to build increasingly complex representation of the input to use for prediction. This approach has transformed whole domains such as computer vision, speech recognition and natural language processing. Despite this success these models have struggled more for structured or tabular data where traditional methods like logistic regression and boosted trees are dominant [8–10].

Recently there have been some advances in developing prediction models for electronic health record data using deep learning [11–13]. The application of attention, an advancement in deep learning, has significantly enhanced various fields including natural language processing and computer vision [14]. Attention is a mechanism which learns the relations between input features and uses them to dynamically focus on specific parts of the input. The recent deep learning EHR models all use different architectures in combination with attention. Choi et al. use recurrent neural networks such as long short-term memory units (LSTM). Kodialam et al. use transformers with and without reverse distillation where their model learns from a linear baseline. Finally, Zhu et al. use graph neural networks (GNN) with graph attention layers in an encoder-decoder setup in combination with variational techniques to regularize the model.

In this study we implement these state-of-the-art EHR models and investigate their performance on three EHR prediction problems and compare against linear and non-linear baseline models. Specifically, we build upon the work of previous literature in structured EHR modelling by reimplementing their model architectures and test thoroughly on a new dataset. The pipeline and models are made publicly available^1^ so others can apply the tools we developed and can test the models on their own OMOP-CDM data.

## Methods

### The dataset

The data used were from the Integrated Primary Care Information (IPCI) database [15]. This is a database including 2.5 million patients from a set of general practitioners (GPs) in the Netherlands and converted to the OMOP-CDM. From the database we extracted three target cohorts (Table 1). We predicted mortality within 30 days in patients over 60 years of age after a GP visit. We predict readmission in 30 days after a hospital discharge in adult patients. Finally, we predict onset of dementia within 5 years from a first GP visit of patients aged 50–79. We selected these problems based on their prevalence in the existing literature and the availability of a sufficient number of outcome events in our data. Mortality and readmission prediction are among the most common prediction problems used in the literature and dementia is common as well [1, 16]. While the data is a GP database certain information from discharge letters is recorded from hospitals, which is especially relevant for the readmission problem. The study was approved by the IPCI supervisory board (approval no. 9/2020 and 2/2022). Comorbidities and more detailed cohort characteristics can be found in supplementary table 1a, 1b and 1c.

**Table 1:** Overview of cohorts used for the prediction models

	Mortality	Readmission		Dementia
Observations	3,836,184 visits	206,995 visits		287,208 patients
Outcome (%)	36,952 (1%)	20,202 (9.8%)		4674 (1.6%)
Sex: # female (%)	2,232,244 (58.2%)	109,684 (53%)		148,660 (51.8%)
Age mean (std)	73.8 (9.0)	62.7 (18.0)		61.3 (8.5)
Index event	GP visit after 60	Inpatient visit of adults		First GP visit of patients aged 50–79
Time-at-risk^1^	30 days	30 days		5 years
Observation window^2^	1 year prior to index	1 year prior to index		1 year prior to index
Features	Age, gender, conditions, drug exposure and procedure codes	Age, gender, conditions, drug exposure and procedure codes		Age, gender, conditions, drug exposure and procedure codes
Number of features	2056	2303		1748
Average features per observation / feature matrix density	49.5 / 2.3%	45.2 / 1.9%		21.8 / 1.2%

^1^Time-at-risk refers to the period after the index event during which predictions are made.

^2^Features are extracted from the observation window

### Preprocessing

Demographics, drug, procedure and condition concept codes were extracted using the OHDSI tool FeatureExtraction^2^. The features were extracted within an observation window starting one year prior to index date until the index date. For the temporal models the input to the model is a sequence of features occurring in the year prior. For models that require static features, three windows were used to aggregate the features across time. The windows reached from 365, 180 and 30 days before and until index. This results in a binary feature indicating whether it was present in the window or not. Features occurring in less than 0.1% of observations were removed. Numerical features were standardized using max absolute scaling to be in the interval [0, 1].

### Model architectures

We developed prediction models using the following model architectures (for more details see the relevant references):

1. RETAIN is a model consisting of two units of recurrent neural network units, in our case LSTMs [11]. First the input goes through a linear embedding layer. The resulting embedding output goes separately through each LSTM. Then attention is added on the hidden states of each LSTM. One uses visit-level attention, and one attends to the coordinate of each visit-level embedding. A context vector is generated from the attentions combined with the visit embeddings, which is then used to make predictions. We made two modifications to the original architecture. First, we used bidirectional LSTMs, which according to a discussion on the author’s GitHub^3^ improved performance, this was also true in our experiments. Second, we concatenated features such as age, sex and visit times to the visit embeddings.
2. The transformer architecture used was based on the implementation in the SARD paper from the authors GitHub^4^. First, an embedding layer calculates the embeddings per feature and sums them up per visit for a visit-level embedding. As in RETAIN we concatenated the non-temporal features, sex and age, to the visit embedding. A sinusoidal temporal embedding was added to the visit embedding. Then the embeddings were fed through standard multi-head attention blocks with layer norm and feed forward networks on top. Finally, the contextualized visit embeddings go through a convolutional prediction head to output the final prediction.
3. For SARD the same architecture as in the transformer is used. First it is trained to match predictions from a linear baseline, then it is finetuned where the supervision is provided by both the labels and linear model predictions using a mixing factor in the loss.
4. The last deep learning model used is a graph neural network from [13]. In contrast to the other deep learning models this model uses non-temporal (static) data. First it embeds the features using a linear embedding layer. Then an encoder consisting of graph attention layers encodes these embeddings into latent variables which are then decoded using a graph attention layer. A node is added to the decoder graph representing the output label and uses its attentions to predict the label. Variational regularization is applied to the latent variables which helps the model learn more expressive representations.
5. A baseline linear model, LASSO was used as implemented in the PLP package [5], it is an L1 regularized logistic regression model which uses adaptive search to select the regularization strength [17].
6. As a second baseline, An XGBoost model was developed using the PLP package. XGBoost is a model known to be very strong in the tabular domain [18].

### Training procedure

A train-test split of 75 − 25% was used for all experiments. For LASSO and XGBoost a three-fold cross validation over the training set was used to select hyperparameters. For the deep learning models a train-validation split of 67 − 33% was used. In mortality and readmission, we divided the data by visit, ensuring that patients were never present across different folds in the process. For dementia the split was by patients. LASSO used the adaptive search, XGBoost used exhaustive grid search, but the deep models used a tree of parzen estimators with 100 iterations over the hyperparameter space [19]. The search space can be seen in supplementary table 2a along with the optimal hyperparameters in supplementary tables 2b to 2e. The deep learning models used a batch size of 512. Early stopping was used with a patience of three epochs and the learning rate was reduced by a factor of ten if the validation loss didn’t improve with patience of one epoch.

For SARD the models were trained to match the LASSO predictions on the training set (distillation) and then finetuned using labels and LASSO predictions (finetuning) on the validation set. Once hyperparameters had been selected then the models were refit on the whole training dataset using the best hyperparameters and tested on the test set. For the deep learning models this included the number of epochs and learning rate schedule.

Since all our prediction problems are imbalanced the deep learning models use a weighted binary cross entropy as objective function where the weight on the positive class is the ratio of negative class observations to positive in the training set. The area under the receiver operating characteristic curve (AUC) and the area under the precision recall curve (AUPRC) were computed for the predictions on the test set. Confidence intervals were computed using the method in Sun et al. [20] for the AUC and from Boyd et al. [21] for the AUPRC.

### Calibration and clinical utility

The deep learning models that employed weighted binary cross-entropy as their objective function required recalibration due to overestimation of risk. The models were recalibrated on the validation set by adding a weight and intercept to the model outputs. These parameters were fit while the rest of the model’s parameter were fixed so that the model produces well calibrated probabilities for the validation set. This is equivalent to Platt’s scaling [22, 23]. For consistency and fairness this procedure was also applied to the baselines. Then to assess calibration set smooth calibration curves were created using restricted cubic splines on the test set.

To assess clinical utility decision curves were computed showing the net benefit of patients for all prediction thresholds [24]. Net benefit is the difference between the proportion of true positives (benefit) and the false positives (harm) weighted by the odds of the selected threshold which would classify someone as high risk. A model with higher net benefit at a given threshold captures more true positives without increasing false positives. The net benefit of the models is compared to the default strategies of treat-all and treat-none.

### Feature importance and hyperparameter analysis

To further gain insight into the specific prediction problems used the top ten LASSO coefficients (by absolute value) were computed. Since an extensive hyperparameter search was performed it allowed for the opportunity to model which hyperparameters are most important to tune for these prediction problems. To model this a random forest model was fit with the hyperparameter combinations as features and the validation AUC as the outcome. Then SHAP was used to compute hyperparameter importance from this model and the results were visualized [25]. This was done for the best deep learning models if they showed benefit over the baselines.

### Reduced feature set

We repeated the analysis above with a reduced feature set of 200 and 20 features. We used the absolute magnitude of coefficients from LASSO to select the largest coefficients and then fit XGBoost, RETAIN, the transformer and SARD to investigate the effect of a reduced feature set on the performance. We did not fit the GNN for this analysis since computationally it was expensive and it was not a best performer in any category.

## Results

### Discrimination

Table 2 shows the discrimination performance for the three prediction problems. SARD performs best both in terms of AUC and AUPRC for the mortality and readmission prediction problems. The LASSO performs best for dementia although the AUC for SARD is very close. XGBoost is in third place. The most significant gains are in the AUPRC for the mortality task where SARD improves on LASSO by 7.3% points and on XGBoost by 0.7% points. Overall, the baselines are quite competitive but the best deep learning methods are comparable or better. XGBoost is better than LASSO on two problems out of three. RETAIN and the GNN struggle to beat the baselines. In all cases, using reverse distillation with SARD yields better results compared to using only the transformer.

**Table 2:** Area under the receiver operating curve (AUC) and area under the precision recall curve (AUPRC) for the models on the three problems. The best performance is in bold

	Mortality		Readmission		Dementia	
	AUC (%)	AUPRC (%)	AUC (%)	AUPRC (%)	AUC (%)	AUPRC (%)
LASSO	92.0 ± 0.33	33.8 ± 0.98	67.0 ± 0.79	18 ± 1.0	**87.5 ± 0.89**	**11.8 ± 2.0**
XGBoost	93.8 ± 0.26	37.9 ± 0.98	67.5 ± 0.79	18.4 ± 1.0	87.2 **±** 0.92	11.6 **±** 2.0
RETAIN	93.6 ± 0.27	34.2 ± 0.98	66.5 ± 0.78	16.9 ± 1.0	85.6 ± 1.06	9.6 ± 2.0
GNN	93.5 ± 0.26	36.7 ± 0.98	65.7 ± 0.79	16.9 ± 1.0	86.5 ± 0.92	8.9 ± 2.0
Transformer	94.1 ± 0.25	37.8 ± 0.98	67.1 ± 0.77	17.7 ± 1.0	87.0 ± 0.91	10.0 ± 2.0
SARD	**94.5 ± 0.24**	**41.1 ± 0.98**	**68.1 ± 0.77**	**18.5 ± 1.0**	87.4 ± 0.90	11.3 ± 2.0

### Calibration

**Fig. 1:**
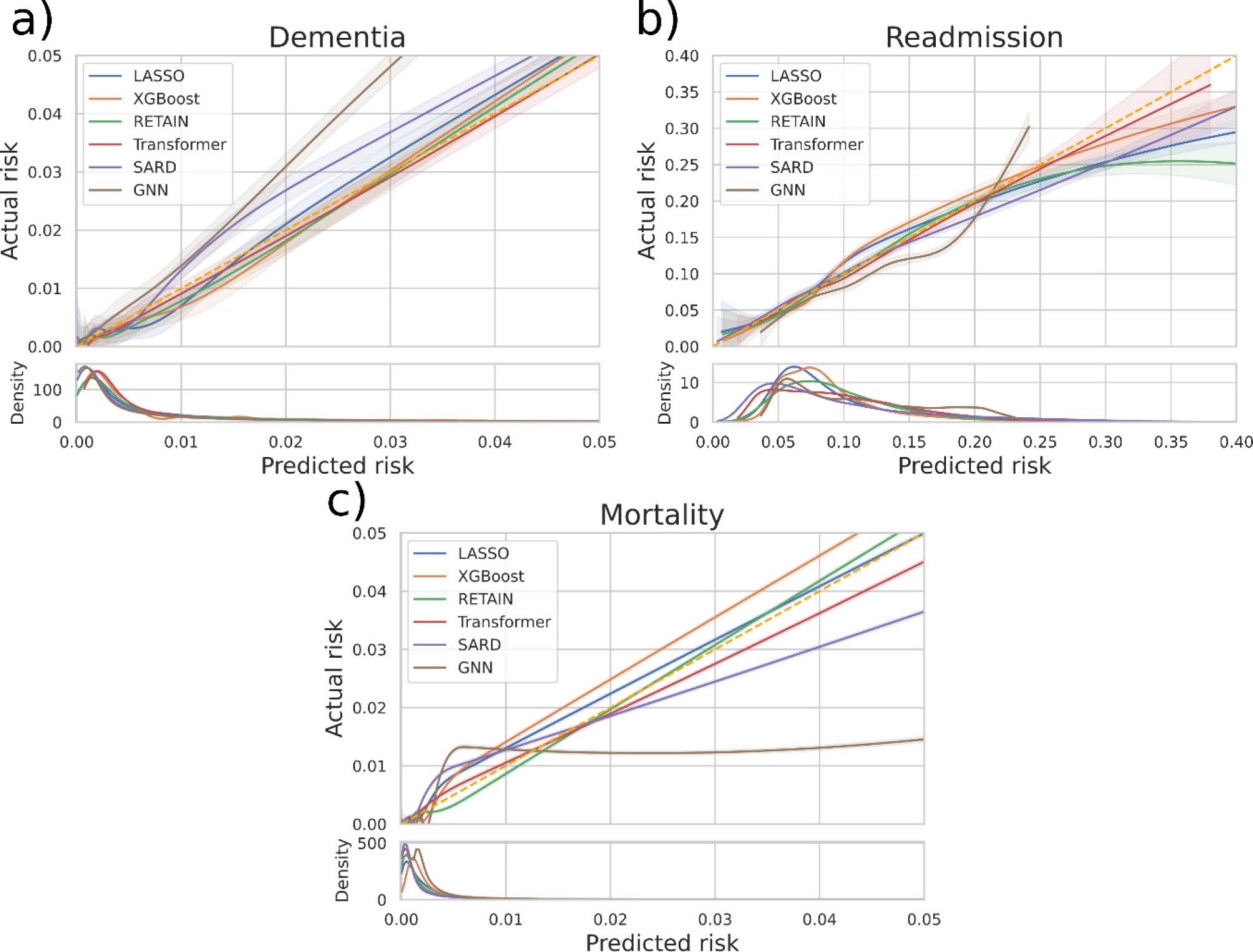
Smooth calibration for the three prediction problems (**a**) Dementia, (**b**) Readmission and (**c**) Mortality. On the x-axis is the predicted risk and, on the y-axis, the actual risk. Below each plot is a density plot showing how the predictions of each model are distributed

The smooth calibration curves for all three problems are depicted in Fig. 1. The plots show that the models are well calibrated for most of their predictions, (see density plots on bottom of Fig. 1) with a few exceptions. For dementia (Fig. 1a) most of the models are well calibrated. Exception is the GNN model which underestimates the risk. SARD underestimates it as well but to a less extent. For readmission (Fig. 1b) LASSO, RETAIN and SARD overestimate risk for high-risk patients but are again well calibrated for most of their predictions. The GNN overestimates risk except for high-risk patients where it underestimates it. For mortality the Transformer and RETAIN are well calibrated while the other models overestimate the risk except for XGBoost which underestimates it.

### Clinical utility

**Fig. 2:**
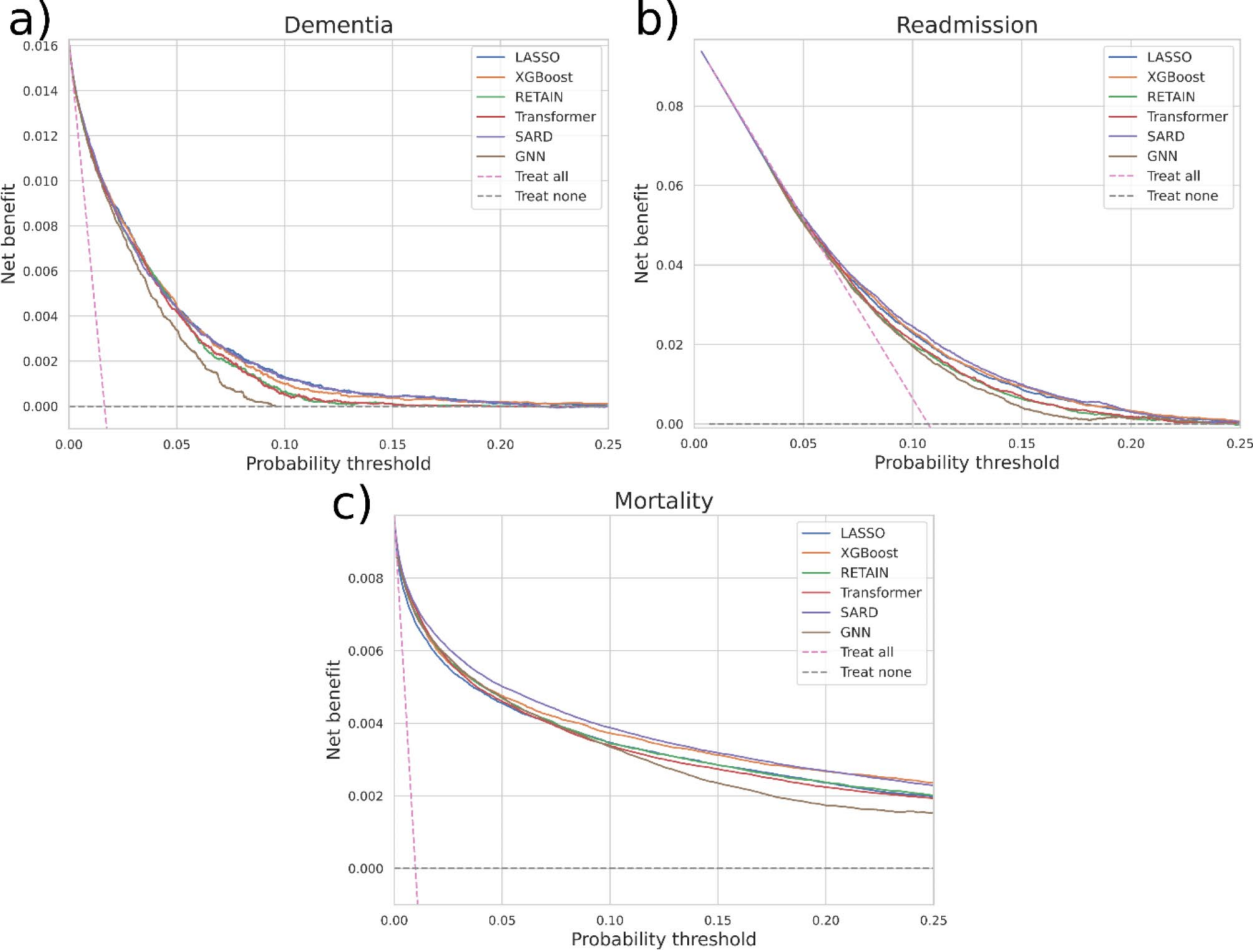
Decision curves showing the net benefit for all the models. It includes the benefit when either treating all or none cases. (**a**) net benefit in for dementia prediction, (**b**) net benefit for readmission prediction and (**c**) net benefit for mortality

The decision curves are plotted in Fig. 2. These show the net benefit over a range of decision thresholds. The net benefit is the benefit due to a decision identifying more true positives without increasing false positives [24, 26]. The net benefit is compared to default strategies of treating all or none. In dementia, SARD and LASSO are equivalent for most of the risk range. There is no benefit over treating none above 20% risk. For both readmission and mortality prediction SARD has the best clinical utility. In all cases there is a range of thresholds where there is more benefit in using a model than treating all/none patients. The threshold signifies the tradeoff between harms due to unnecessary treatment and missing a true positive and needs to be selected using clinical knowledge depending on the specific problem.

### Feature importance

Feature importance of LASSO for the three prediction problems can be seen in supplementary Table 3. Displayed are coefficients with the largest absolute value. In mortality most of the largest coefficients are negative, indicating the model is learning things that are predictive of surviving. However, the largest coefficient is age and is positive. For readmission many of the features have to do with cancers of various kinds. For dementia age is the largest with various drugs and conditions with positive coefficients. These include vitamins such as D-vitamin and B-vitamin, but deficiencies of those vitamins have been linked to increased dementia risk [27, 28] and injuries such as head injury are as well linked to increased dementia risk [29]. Positive coefficients for the vitamins could be explained by doctors prescribing these as a preventative measure to patients with subclinical presentation of symptoms.

**Fig. 3:**
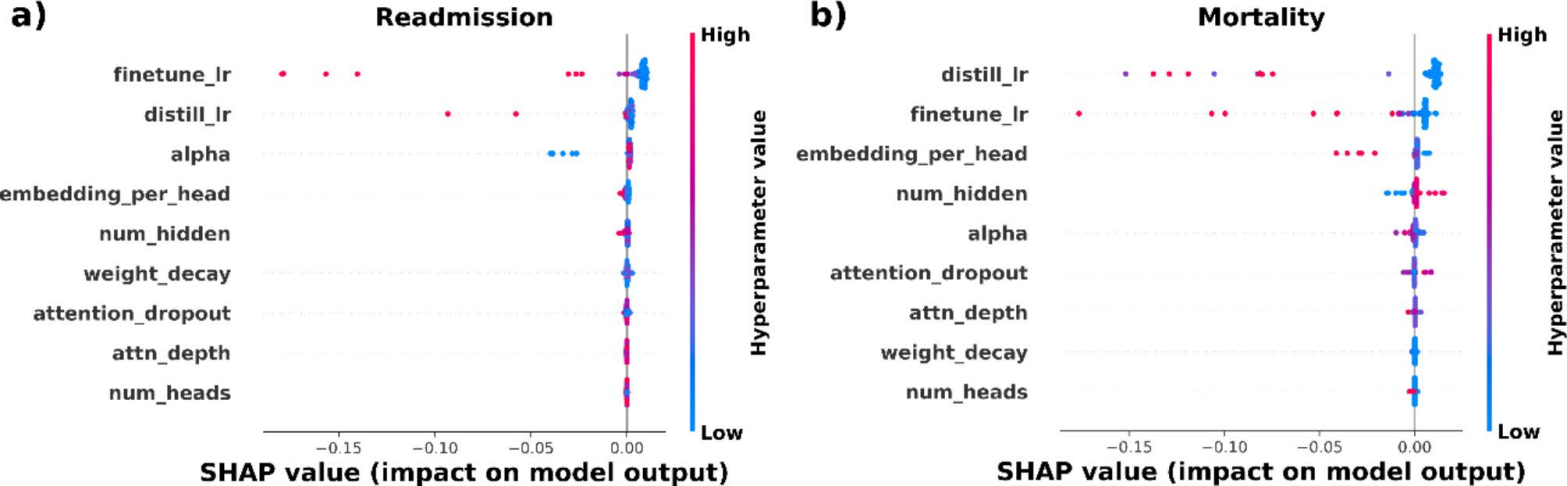
Feature importance for (**a**) readmission and (**b**) mortality. On y-axis are the hyperparameter and on the x-axis are the effects of those on the model output (validation AUC). Red color means higher values of the hyperparameter and blue is lower. lr: learning rates, num_head: number of attention heads, num_hidden: number of neurons in fully connected layers, attn_depth: number of attention layers

In Fig. 3 we see the importance of the hyperparameters for SARD. We focus here on the problems where the performance was better than the baselines (readmission and mortality prediction). The other models are in supplementary Fig. 1 to 4. The two most important hyperparameters are the different learning rates while the others vary by prediction problem. Lower learning rates improve the predictions. The best performing deep learning models seem to prefer lower embedding sizes (see supplementary Table 2c).

### Reduced feature set

The results for the discrimination performance of the reduced feature set can been seen in supplementary Tables 4 and 5. There is a difference between problems with how affected the performance is using a reduced set. The dementia problem is barely affected while readmission is moderately affected. Mortality problem shows the greatest drop in performance. Interestingly for readmission and mortality the non-deep learning baselines suffer more drop as the feature set is reduced.

## Discussion

In this study we empirically investigated some of the state-of-the-art approaches in supervised learning using neural networks combined with attention. Our results show that using deep learning with attention can outperform strong baselines. Overall however, good baselines are still competitive. Using a linear baseline as a teacher model in a distillation setting always improves the deep learning model and depending on the problem might provide additional benefit over the linear model. For readmission and dementia problem most models are decently calibrated except for the GNN model. For mortality there are signs of overestimating risk for LASSO, SARD and the GNN and underestimation for XGBoost. In terms of clinical utility SARD has higher net benefit on readmission and mortality over LASSO and XGBoost. To ensure good performance a careful tuning of the learning rate is required for the deep learning models.

In terms of discrimination SARD has the best performance of two out of three prediction problems. The performance difference between SARD and the baselines was greatest on the mortality problem which had the largest cohort size. The differences we find are more modest than many reported in the literature. The biggest difference between a deep learning model using attention and logistic regression is 15–25% points from Solares et al. [30]. The best performing model in their study was RETAIN. One thing to note that was different in their study than ours is that they reduced the high dimensionality from thousands down to 326 by grouping medical codes. Choi et al. [11] founds differences of 7% points between RETAIN and logistic regression, which is less than Solares et al. but more than Kodialam et al. [12]. However, in their original study they only train RETAIN on medical codes but no other data that is usually available and can improve performance such as demographics. Our results do agree broadly with Kodialam et al. and Rajkomar et al. [31] where improvements in discrimination are smaller, on the order of few percentage points.

The deep learning models seem to be more robust to a set of reduced features (supplementary Tables 4 and 5), in particular in readmission and mortality. However, it is important to note that the best performing models overall are always those with the full feature set. If there are few features available, it might give greater gains to use the deep learning models. This might also explain why some of the previous literature finds greater gains using different feature settings. However if you have more features available that is the best pathway to improve the performance. Although deep learning research rarely addresses the issue of calibration, it is crucial in safety-critical domains such as medicine to ensure that a model’s risk estimates are appropriately calibrated for effective medical decision-making [32]. Our analysis indicates that, except for the GNN that utilizes variational techniques, the deep learning models exhibit similar levels of calibration as the baseline methods. However, it is essential to assess calibration on a problem-by-problem basis, as we found that SARD and LASSO tend to overestimate the risk for the mortality prediction task, which suggests overfitting. In literature it is common to use weighting in the objective function for problems where the outcome is rare. This guarantees that the model will be miscalibrated and overestimates risk. Such a model needs to be recalibrated before use.

The assessment of clinical utility is crucial for clinical prediction models. One approach to evaluating clinical utility is through decision curve analysis, which has been overlooked in the deep learning literature. While models may demonstrate similar discrimination performance as measured by the AUC or AUPRC, differences in clinical utility across the thresholds used in practice can exist [26]. SARD not only improved overall discrimination in two problems, but also demonstrated enhanced clinical utility over the baselines. Specifically, SARD was able to identify more true positives without increasing the number of false positives, suggesting potential benefits without added harm. However, the clinical relevance of these findings will ultimately depend on the specific context in which the model is being used, including the decisions to be made, the associated costs, and the potential benefits.

Medical problems in real-life often involve rare outcomes, with outcome rates typically ranging from 1 to 10% in our three cases. In such situations, relying solely on the AUC may not be sensitive enough to the absolute number of false positives relative to true positives. Thus, a high AUC can be achieved even when a model has a significantly higher number of false positives than true positives. To capture this behavior more accurately, the AUPRC is a more sensitive measure [33]. Notably, for mortality prediction, our deep learning models demonstrated the greatest improvement in AUPRC. This finding suggests that, on average, these models achieve higher precision and a higher ratio of true positives to positive predictions. In clinical practice, this metric is important because it is closely related to the concept of alarm fatigue [34]. Alarm fatigue occurs when clinical staff are constantly inundated with unreliable or unactionable alarms. A prediction model with low precision can produce such alarms, so it is crucial to utilize a metric that accurately captures this situation.

The feature importance analysis indicated that the learning rates are the most important hyperparameters for the models. Overall, the best performing models preferred lower embedding sizes indicating that the features are compressed quite a bit to reduce overfitting. For the mortality problem, a lower alpha parameter was preferred when blending losses during reverse distillation. This suggests that the model only required initialization to match the performance of the linear model and subsequently relied less on its input. This was not the case for readmission where higher alpha values were preferred. This contrasts with the original paper where lower alpha values, even as low as zero, are preferred [12]. Higher alpha values could mean that there is more tendency to overfit and the model is using the linear model loss as regularization. By having high alpha values, the model is constraining itself to not deviate much from the linear model. Looking at the hyperparameter importance for the other models (supplementary Figs. 1–4) the learning rates seem to have most affect on the model output. Other important hyperparameter depend on the model but parameters such as embedding size, layer number and dimension are as well important.

There are some limitations to our study. First, we only assess internal validity. To develop a robust clinical prediction model it is important to assess generalizability and reliability of predictions to slightly different populations through external validation [35]. Second, although we use three different problems it is all from the same database. To be able to generalize the results it is important to replicate them across different databases. Different database characteristics might already explain the difference of our results to some of the literature. The prediction problems we chose might themselves have their own limitations, for example dementia diagnosis is used to define the outcome in one problem but dementia has a significant rate of underdiagnosis [36]. However, we believe the algorithms are affected by this equally and that this does not affect our comparison. Because of our data we only had available three prediction problems to test on. This precludes testing of statistical significance using appropriate tests such as a Friedman’s test and a critical difference diagram. Finally, we did not include labs/measurements as features. This was done to simplify the comparison since measurements have missing values that need to be accounted for. Most of the compared models did not use these features in their original papers. However, we believe using these features could improve the deep learning models considerably and this should be considered in future work.

## Conclusion

In this paper we implement the state-of-the art approaches using attention based deep neural networks for supervised learning. We developed these models on the OMOP-CDM which allows others to reuse our code and replicate our findings. The study shows that deep learning with attention can outperform strong baselines in supervised learning, but performance depends on the problem. In terms of calibration, most deep learning models exhibit similar levels as the baseline methods except for the GNN model. SARD demonstrates enhanced clinical utility over LASSO and XGBoost for readmission and mortality prediction.

### Electronic supplementary material

Below is the link to the electronic supplementary material.

Supplementary Material 1

## Data Availability

No individual participant data can be shared for privacy reasons. The code and models are available on github^5^. Aggregated data not shared there are available upon a reasonable request. Requests should be directed to the corresponding author.

## Footnotes

1 https://github.com/mi-erasmusmc/DeepLearningEHR.

2 https://ohdsi.github.io/FeatureExtraction/.

3 https://github.com/mp2893/retain.

4 https://github.com/clinicalml/omop-learn.
