# Supplementary material for: Attention-based neural networks for clinical prediction modelling on electronic health records
Source: BMC Med Res Methodol. 2023 Dec 7;23:285. doi: 10.1186/s12874-023-02112-2 (PMC10701944; doi:10.1186/s12874-023-02112-2)
Supplement: Supplementary file 1 — Supplementary Material 1 [file 12874_2023_2112_MOESM1_ESM.docx]

**SUPPLEMENTARY MATERIAL**

**Supplementary table 1a: Mortality cohort characteristics**

| **Characteristic** | **Counts (%) (n = 3,836,184)** | **Characteristic** | **% (n = 3,836,184)** |
| --- | --- | --- | --- |
| **Age group** |  | **Medical history: Cardiovascular disease** |  |
| 60 - 64 | 700,075 (18.2%) | Atrial fibrillation | 102,040 (2.7%) |
| 65 - 69 | 723,190 (18.9%) | Cerebrovascular disease | 98,875 (2.6%) |
| 70 - 74 | 728,497 (19%) | Coronary arteriosclerosis | 9,332 (0.2%) |
| 75 - 79 | 618,669 (16.1%) | Heart disease | 376,771 (9.8%) |
| 80 - 84 | 511,650 (13.3%) | Heart failure | 104,269 (2.7%) |
| 85 - 89 | 351,057 (9.2%) | Ischemic heart disease | 119,952 (3.1%) |
| 90 - 94 | 160,037 (4.2%) | Peripheral vascular disease | 64 (0%) |
| 95 - 99 | 38,154 (1%) | Pulmonary embolism | 12,593 (0.3%) |
| 100 - 104 | 4,451 (0.1%) | Venous thrombosis | 31,889 (0.8%) |
| 105 - 109 | 404 (0%) | **Medical history: Neoplasms** |  |
| **Gender: female** | 2,232,244 (58.2%) | Hematologic neoplasm | 5,134 (0.1%) |
| **Medical history: General** |  | Malignant lymphoma | 4,555 (0.1%) |
| Acute respiratory disease | 476,146 (12.4%) | Malignant neoplastic disease | 263,574 (6.9%) |
| Attention deficit hyperactivity disorder | 1,196 (0%) | Malignant tumor of breast | 23,361 (0.6%) |
| Chronic liver disease | 1,595 (0%) | Malignant tumor of lung | 24,277 (0.6%) |
| Chronic obstructive lung disease | 151,377 (3.9%) | Malignant tumor of urinary bladder | 10,663 (0.3%) |
| Crohn's disease | 6,193 (0.2%) | **Medication use** |  |
| Dementia | 66,260 (1.7%) | Agents acting on the renin-angiotensin system | 1,675,231 (43.7%) |
| Depressive disorder | 61,561 (1.6%) | Antibacterials for systemic use | 1,898,217 (49.5%) |
| Diabetes mellitus | 368,523 (9.6%) | Antidepressants | 541,871 (14.1%) |
| Gastroesophageal reflux disease | 21,558 (0.6%) | Antiepileptics | 237,990 (6.2%) |
| Gastrointestinal hemorrhage | 61,002 (1.6%) | Antiinflammatory and antirheumatic products | 1,378,419 (35.9%) |
| Human immunodeficiency virus infection | 129 (0%) | Antineoplastic agents | 186,282 (4.9%) |
| Hyperlipidemia | 697,35 (1.8%) | Antipsoriatics | 35,060 (0.9%) |
| Hypertensive disorder | 478,967 (12.5%) | Antithrombotic agents | 1,330,538 (34.7%) |
| Lesion of liver | 3,858 (0.1%) | Beta blocking agents | 1,366,823 (35.6%) |
| Obesity | 22,994 (0.6%) | Calcium channel blockers | 825,455 (21.5%) |
| Osteoarthritis | 275,017 (7.2%) | Diuretics | 1,401,960 (36.5%) |
| Pneumonia | 193,176 (5%) | Drugs for acid related disorders | 2,191,094 (57.1%) |
| Psoriasis | 22,574 (0.6%) | Drugs for obstructive airway diseases | 1,590,753 (41.5%) |
| Renal impairment | 121,753 (3.2%) | Drugs used in diabetes | 730,857 (19.1%) |
| Rheumatoid arthritis | 16,347 (0.4%) | Immunosuppressants | 92,739 (2.4%) |
| Schizophrenia | 1,852 (0%) | Lipid modifying agents | 1,659,634 (43.3%) |
| Ulcerative colitis | 5,757 (0.2%) | Opioids | 991,831 (25.9%) |
| Urinary tract infectious disease | 441,104 (11.5%) | Psycholeptics | 1,041,992 (27.2%) |
| Viral hepatitis C | 264 (0%) | Psychostimulants, agents used for adhd and nootropics | 13,793 (0.4%) |
| Visual system disorder | 607,663 (15.8%) |  |  |

**Supplementary table 1b: Readmission cohort characteristics**

| **Characteristic** | **Counts (%) (n = 206,995)** | **Characteristic** | **% (n = 206,995)** |
| --- | --- | --- | --- |
| **Age group** |  | **Medical history: Cardiovascular disease** |  |
| 15 - 19 | 1,863 (0.9%) | Atrial fibrillation | 6,493 (3.4%) |
| 20 - 24 | 5,032 (2.4%) | Cerebrovascular disease | 8,894 (4.6%) |
| 25 - 29 | 6,501 (3.1%) | Coronary arteriosclerosis | 759 (0.4%) |
| 30 - 34 | 7,285 (3.5%) | Heart disease | 26,716 (14.1%) |
| 35 - 39 | 7,337 (3.5%) | Heart failure | 8,027 (4.0%) |
| 40 - 44 | 8,031 (4.0%) | Ischemic heart disease | 9,740 (5.5%) |
| 45 - 49 | 10,807 (5.4%) | Peripheral vascular disease | 87 (0.0%) |
| 50 - 54 | 13,679 (6.8%) | Pulmonary embolism | 1,708 (1.0%) |
| 55 - 59 | 16,567 (8.2%) | Venous thrombosis | 2,253 (1.1%) |
| 60 - 64 | 19,387 (9.6%) | **Medical history: Neoplasms** |  |
| 65 - 69 | 22,271 (11.1%) | Hematologic neoplasm | 660 (0.3%) |
| 70 - 74 | 24,819 (12.1%) | Malignant lymphoma | 774 (0.4%) |
| 75 - 79 | 23,655 (11.3%) | Malignant neoplastic disease | 27,475 (13.4%) |
| 80 - 84 | 19,912 (9.4%) | Malignant tumor of breast | 3,856 (1.9%) |
| 85 - 89 | 13,309 (6.1%) | Malignant tumor of lung | 3,402 (1.6%) |
| 90 - 94 | 5,423 (2.4%) | Malignant tumor of urinary bladder | 2,013 (1.1%) |
| 95 - 99 | 1,028 (0.4%) | **Medication use** |  |
| **Gender: female** | 109727 (53%) | Agents acting on the renin-angiotensin system | 68,424 (34.2%) |
| **Medical history: General** |  | Antibacterials for systemic use | 99,177 (50.9%) |
| Acute respiratory disease | 24,679 (12.4%) | Antidepressants | 29,219 (14.5%) |
| Attention deficit hyperactivity disorder | 531 (0.3%) | Antiepileptics | 15,514 (7.7%) |
| Chronic liver disease | 228 (0.1%) | Antiinflammatory and antirheumatic products | 77,110 (40.8%) |
| Chronic obstructive lung disease | 9,019 (4.6%) | Antineoplastic agents | 10,295 (5.6%) |
| Crohn's disease | 760 (0.4%) | Antipsoriatics | 11,318 (1.0%) |
| Dementia | 1,999 (1%) | Antithrombotic agents | 67,501 (35.7%) |
| Depressive disorder | 4,616 (2.2%) | Beta blocking agents | 62,340 (31.3%) |
| Diabetes mellitus | 14,252 (7.3%) | Calcium channel blockers | 35,431 (17.6%) |
| Gastroesophageal reflux disease | 1,008 (0.5%) | Diuretics | 59,630 (29.7%) |
| Gastrointestinal hemorrhage | 4,693 (2.3%) | Drugs for acid related disorders | 109,531 (55.9%) |
| Human immunodeficiency virus infection | 51 (0.0%) | Drugs for obstructive airway diseases | 77,064 (38.0%) |
| Hyperlipidemia | 2,069 (1.1%) | Drugs used in diabetes | 30,295 (14.6%) |
| Hypertensive disorder | 15,408 (7.9%) | Immunosuppressants | 6,705 (3.2%) |
| Lesion of liver | 600 (0.3%) | Lipid modifying agents | 65,346 (31.6%) |
| Obesity | 1,920 (1.0%) | Opioids | 58,738 (28.4%) |
| Osteoarthritis | 1,3124 (6.7%) | Psycholeptics | 56,142 (27.1%) |
| Pneumonia | 12,715 (6.5%) | Psychostimulants, agents used for adhd and nootropics | 1,867 (0.9%) |
| Psoriasis | 1,140 (0.6%) |  |  |
| Renal impairment | 6,093 (3.0%) |  |  |
| Rheumatoid arthritis | 854 (0.5%) |  |  |
| Schizophrenia | 212 (0.1%) |  |  |
| Ulcerative colitis | 630 (0.3%) |  |  |
| Urinary tract infectious disease | 21,867 (10.5%) |  |  |
| Viral hepatitis C | 28 (0.0%) |  |  |
| Visual system disorder | 24,451 (11.8%) |  |  |

**Supplementary table 1c: Dementia cohort characteristics:**

| **Characteristic** | **Counts (%) (n = 287,208)** | **Characteristic** | **% (n = 287,208)** |
| --- | --- | --- | --- |
| **Age group** |  | **Medical history: Cardiovascular disease** |  |
| 50 - 54 | 80,361 (28%) | Atrial fibrillation | 4,227 (1.5%) |
| 55 - 59 | 52,189 (18.2%) | Cerebrovascular disease | 4,566 (1.6%) |
| 60 – 64 | 50,571 (17.6%) | Coronary arteriosclerosis | 796 (0.3%) |
| 65 – 69 | 45,425 (15.8%) | Heart disease | 19,328 (6.7%) |
| 70 - 74 | 34,009 (11.8%) | Heart failure | 1,470 (0.5%) |
| 75 - 79 | 24,653 (8.6%) | Ischemic heart disease | 9,888 (3.4%) |
| **Gender: female** | 148,660 (51.8%) | Peripheral vascular disease | 2 (0%) |
| **Medical history: General** |  | Pulmonary embolism | 710 (0.2%) |
| Acute respiratory disease | 33,971 (11.8%) | Venous thrombosis | 1,945 (0.7%) |
| Attention deficit hyperactivity disorder | 282 (0.1%) | **Medical history: Neoplasms** |  |
| Chronic liver disease | 74 (0.0%) | Hematologic neoplasm | 241 (0.1%) |
| Chronic obstructive lung disease | 8190 (2.9%) | Malignant lymphoma | 282 (0.1%) |
| Crohn's disease | 705 (0.2%) | Malignant neoplastic disease | 11,867 (4.1%) |
| Dementia | 0 (0.0%) | Malignant tumor of breast | 2636 (0.9%) |
| Depressive disorder | 7,371 (2.6%) | Malignant tumor of lung | 339 (0.1%) |
| Diabetes mellitus | 22,533 (7.8%) | Malignant tumor of urinary bladder | 442 (0.2%) |
| Gastroesophageal reflux disease | 2,477 (0.9%) | **Medication use** |  |
| Gastrointestinal hemorrhage | 2,562 (0.9%) | Agents acting on the renin-angiotensin system | 68,084 (23.7%) |
| Human immunodeficiency virus infection | 0 (0.0%) | Antibacterials for systemic use | 92,804 (32.3%) |
| Hyperlipidemia | 13,164 (4.6%) | Antidepressants | 25,622 (8.9%) |
| Hypertensive disorder | 50,595 (17.6%) | Antiepileptics | 7,502 (2.6%) |
| Lesion of liver | 24 (0.0%) | Antiinflammatory and antirheumatic products | 85,462 (29.8%) |
| Obesity | 2,218 (0.8%) | Antineoplastic agents | 5,513 (1.9%) |
| Osteoarthritis | 15,183 (5.3%) | Antipsoriatics | 2,002 (0.7%) |
| Pneumonia | 5,555 (1.9%) | Antithrombotic agents | 36,450 (12.7%) |
| Psoriasis | 3,022 (1.1%) | Beta blocking agents | 57,588 (20.1%) |
| Renal impairment | 2,217 (0.8%) | Calcium channel blockers | 26,625 (9.3%) |
| Rheumatoid arthritis | 2,112 (0.7%) | Diuretics | 54,244 (18.9%) |
| Schizophrenia | 278 (0.1%) | Drugs for acid related disorders | 90,186 (31.4%) |
| Ulcerative colitis | 725 (0.3%) | Drugs for obstructive airway diseases | 82,359 (28.7%) |
| Urinary tract infectious disease | 17,321 (6.0%) | Drugs used in diabetes | 26,819 (9.3%) |
| Viral hepatitis C | 49 (0.0%) | Immunosuppressants | 3,474 (1.2%) |
| Visual system disorder | 31,918 (11.1%) | Lipid modifying agents | 70,450 (24.5%) |
|  |  | Opioids | 41,116 (14.3%) |
|  |  | Psycholeptics | 46,585 (16.2%) |
|  |  | Psychostimulants, agents used for adhd and nootropics | 1,263 (0.4%) |

**Supplementary table 2a: Hyperparameter space deep learning models**

| RETAIN | | Transformer | | SARD* | | GNN | |
| --- | --- | --- | --- | --- | --- | --- | --- |
| Embedding dimension: | 32 to 256, increments of 16 | Embedding per attention head: | 16 to 96, increments of 16 | Distill learning rate: | 1e-5 to 1e-3 | Embedding dimension | 32 to 1024 |
| Embedding dropout: | 0 to 0.3 uniform distribution | Attention heads: | 2 to 6, increments of 2 | Finetune learning rate: | 1e-5 to 1e-3 | Dropout | 0 to 0.3 |
| Alpha size: | 32 to 256, increments of 16 | Number of layers: | 1 to 6, increments of 1 | Alpha: | 0 to 1, increments of 0.05 | Number of layers | 1 to 6 |
| Beta size: | 32 to 256, increments of 16 | Size of hidden layers: | 256 to 2048, increments of 1 |  |  | Number of heads | 1 to 4 |
| Context dropout: | 0 to 0.3, increments of 0.05 | Attention dropout: | 0 to 0.3 increments of 0.05 |  |  | Attention dropout | 0 to 0.3 |
| Number of layers: | 1 to 8 | Residual dropout: | 0 to 0.3 increments of 0.05 |  |  | Learning Rate | 1-e5 to 1e-2 log-uniform distribution |
| Learning rate: | 1e-5 to 1e-1 log-uniform distribution | Feedforward dropout: | 0 to 0.3 increments of 0.05 |  |  | Weight decay | 1e-8 to 1e-3 log-uniform distribution |
| Weight decay: | 1e-8 to 1e-3 log-uniform distribution | Learning rate: | 1e-5 to 1e-1 log-uniform distribution |  |  |  |  |
|  |  | Weight Decay: | 1e-8 to 1e-3 log-uniform distribution |  |  |  |  |

*SARD has same hyperparameters as the transformer as well as these additional ones

**Supplementary table 2b: Best hyperparameters for RETAIN:**

| RETAIN | Mortality | Readmission | Dementia |
| --- | --- | --- | --- |
| Embedding dimension: | 96 | 192 | 32 |
| Embedding dropout: | 0.3 | 0.01 | 0.13 |
| Alpha size: | 32 | 32 | 224 |
| Beta size: | 64 | 96 | 64 |
| Context dropout: | 0 | 0.14 | 0.05 |
| Number of layers: | 1 | 4 | 1 |
| Learning rate: | 9.1e-4 | 1.1e-3 | 4e-3 |
| Weight decay: | 4.5e-5 | 8.3e-6 | 9.5e-4 |

**Supplementary table 2c: Best hyperparameters for Transformer/SARD**

| Transformer / SARD | Mortality | Readmission | Dementia |
| --- | --- | --- | --- |
| Embedding dimension | 60 | 92 | 92 |
| Attention heads | 2 | 6 | 4 |
| Number of layers | 3 | 4 | 6 |
| Hidden layer size | 1828 | 651 | 1183 |
| Attention dropout | 0.15 | 0.2 | 0.25 |
| Residual dropout | 0.15 | 0.2 | 0.25 |
| Feedforward dropout | 0.15 | 0.2 | 0.25 |
| Learning rate† | 5.2e-5 | 1.4e-3 | 5e-4 |
| Weight decay | 1.3e-7 | 1.3e-4 | 4.7e-5 |
| Distill learning rate* | 3e-5 | 6.7e-4 | 5e-3 |
| Finetune learning rate* | 4.5e-5 | 3.6e-4 | 2.2e-5 |
| Alpha* | 0.2 | 0.35 | 0.6 |

*Only for SARD, †Only for Transformer

**Supplementary table 2d: Best hyperparameters for the GNN**

| GNN | Mortality | Readmission | Dementia |
| --- | --- | --- | --- |
| Embedding dimension: | 108 | 490 | 842 |
| Number of layers: | 2 | 5 | 4 |
| Number of heads: | 4 | 3 | 3 |
| Dropout: | 0.005 | 0.001 | 0.16 |
| Attention dropout: | 0.20 | 0.28 | 0.25 |
| Learning rate: | 2.6e-4 | 1.5e-5 | 3e-3 |
| Weight decay: | 7.6e-6 | 6.8e-4 | 6.5e-7 |

**Supplementary data 1**

**Hyperparameter space for LASSO:**

Initial variance: 0.01

**Best hyperparameters for LASSO:**

Mortality: variance of 1.44, Readmission: variance of 0.037, Dementia: variance of 0.048

Final model sizes: mortality 3178 nonzero coefficients, readmission 1224, dementia 369

**Hyperparameter space for XGBoost:**

Number of trees: (100, 300)

Max depth: (4, 6, 8)

Learning rate: (0.05, 0.1, 0.3)

Early stopping rounds: 25

Lambda: 1

**Supplementary table 2e: Best hyperparameters for XGBoost**

| XGBoost | Mortality | Readmission | Dementia |
| --- | --- | --- | --- |
| Number of trees: | 300 | 300 | 300 |
| Max depth: | 8 | 4 | 4 |
| Learning rate: | 0.05 | 0.1 | 0.1 |

**Supplementary table 3: Top ten features selected by LASSO**

|  | **Mortality** | | **Readmission** | | **Dementia** | |
| --- | --- | --- | --- | --- | --- | --- |
|  | **Feature** | **Coefficient value** | **Feature** | **Coefficient value** | **Feature** | **Coefficient value** |
| 1. | Age in years | 5.54 | Condition: Malignant tumor of cervix 180 days prior | 1.25 | Age in years | 13.7 |
| 2. | Drug: Cholestyramine Resin 365 days prior | -4.94 | Procedure: Radiographic imaging procedure 365 days prior | -1.17 | Drug: Cholecalciferol in 365 days prior | 1.46 |
| 3. | Drug: Cholestyramine Resin 180 days prior | 4.92 | Drug: Dexamethasone 30 days prior | 0.99 | Drug: Haloperidol 365 days prior | 1.0 |
| 4. | Condition: Muscle finding 30 days prior | -3.35 | Condition: Malignant neoplasm of nervous system 180 days prior | 0.81 | Drug: Cholecalciferol 180 days prior | 0.99 |
| 5. | Drug: Sildenafil 180 days prior | -3.24 | Condition: Malignant neoplastic disease 365 days prior | 0.81 | Condition: General health deterioration 365 days prior | 0.87 |
| 6. | Drug: Estradiol 30 days prior | -2.74 | Condition: Leukemia 365 days prior | 0.78 | Condition: Disability 365 days prior | 0.57 |
| 7. | Condition: Injury of eye region 365 days prior | -2.71 | Condition: Malignant tumor of stomach 365 days prior | 0.78 | Condition: Injury of head 365 days prior | 0.57 |
| 8. | Drug: Duloxetine 365 days prior | -2.56 | Condition: Malignant tumor of biliary tract 365 days prior | 0.74 | Drug: Thiamine 365 days prior | 0.54 |
| 9. | Drug: Candesartan cilexetil/ hydrochlorothiazide 30 days prior | -2.52 | Drug: Aprepitant 30 days prior | 0.74 | Drug: Venlafaxine 365 days prior | 0.47 |
| 10. | Procedure: Cytopathology procedure, preparation of smear, genital source 180 days prior | -2.45 | Age in years | 0.74 | Condition: Bacterial conjunctivitis 365 days prior | 0.45 |

**Supplementary table 4: Discrimination performance with top 200 features**

|  | Mortality | | Readmission | | Dementia | |
| --- | --- | --- | --- | --- | --- | --- |
|  |  |  |  |  |  |  |
|  | AUC (%) | AUPRC (%) | AUC (%) | AUPRC (%) | AUC (%) | AUPRC (%) |
| LASSO | 83.17±0.48 | 23.97±0.98 | 63.84±0.83 | 16.57±1.0 | 86.9±0.90 | 9.9±2.0 |
| XGBoost | 83.9±0.48 | 26.36±0.98 | 64.85±0.81 | 17.51±1.0 | 86.7±0.93 | 9.8±2.0 |
| RETAIN | 85.33±0.48 | 18.69±0.98 | 62.5±0.78 | 14.24±1.0 | 85.6±0.91 | 9.2±2.0 |
| Transformer | 87.66±0.48 | 24±0.98 | 64.36±0.78 | 15.58±1.0 | 86.7±0.91 | 9.6±2.0 |
| SARD | 87.81±0.39 | 26.31±0.98 | 64.33±0.78 | 15.58±1.0 | 87.01±0.90 | 10.20±2.0 |

**Supplementary table 5: Discrimination performance with top 20 features**

|  | Mortality | | Readmission | | Dementia | |
| --- | --- | --- | --- | --- | --- | --- |
|  |  |  |  |  |  |  |
|  | AUC (%) | AUPRC (%) | AUC (%) | AUPRC (%) | AUC (%) | AUPRC (%) |
| LASSO | 69.78±0.48 | 2.85±0.98 | 58.55±0.82 | 14±1.0 | 86.7±0.90 | 9.1±2.0 |
| XGBoost | 69.62±0.56 | 2.76±0.98 | 59.47±0.83 | 14.9±1.0 | 86.8±0.91 | 9.2±2.0 |
| RETAIN | 75.64±0.56 | 7.04±0.98 | 57±0.80 | 11.4±1.0 | 85.7±0.90 | 9.1±2.0 |
| Transformer | 75.89±0.55 | 10.6±0.98 | 60.41±0.83 | 14.76±1.0 | 86.7±0.90 | 9±2.0 |
| SARD | 76.34±0.54 | 11.56±0.98 | 60.47±0.83 | 14.98±1.0 | 86.8±0.90 | 9.4±2.0 |


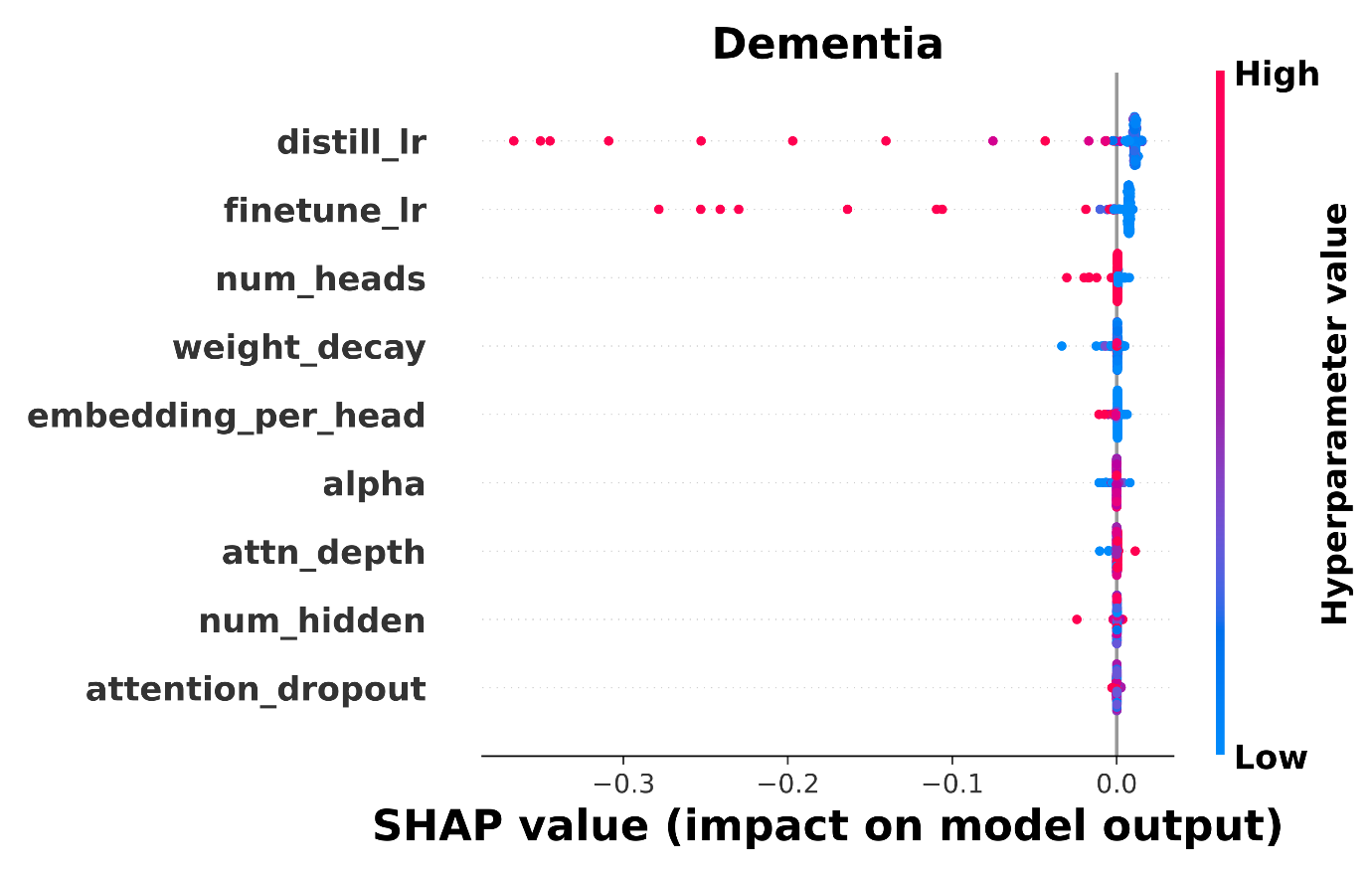


**Supplementary Figure 1**: Feature importances for SARD hyperparameters for Dementia prediction problem. On the y-axis are the hyperparameters while in the x-axis is the impact of them on the model output (validation AUC). Red means higher values of the hyperparameter while blue means lower. lr: learning rate, dim_emb: embedding dimension


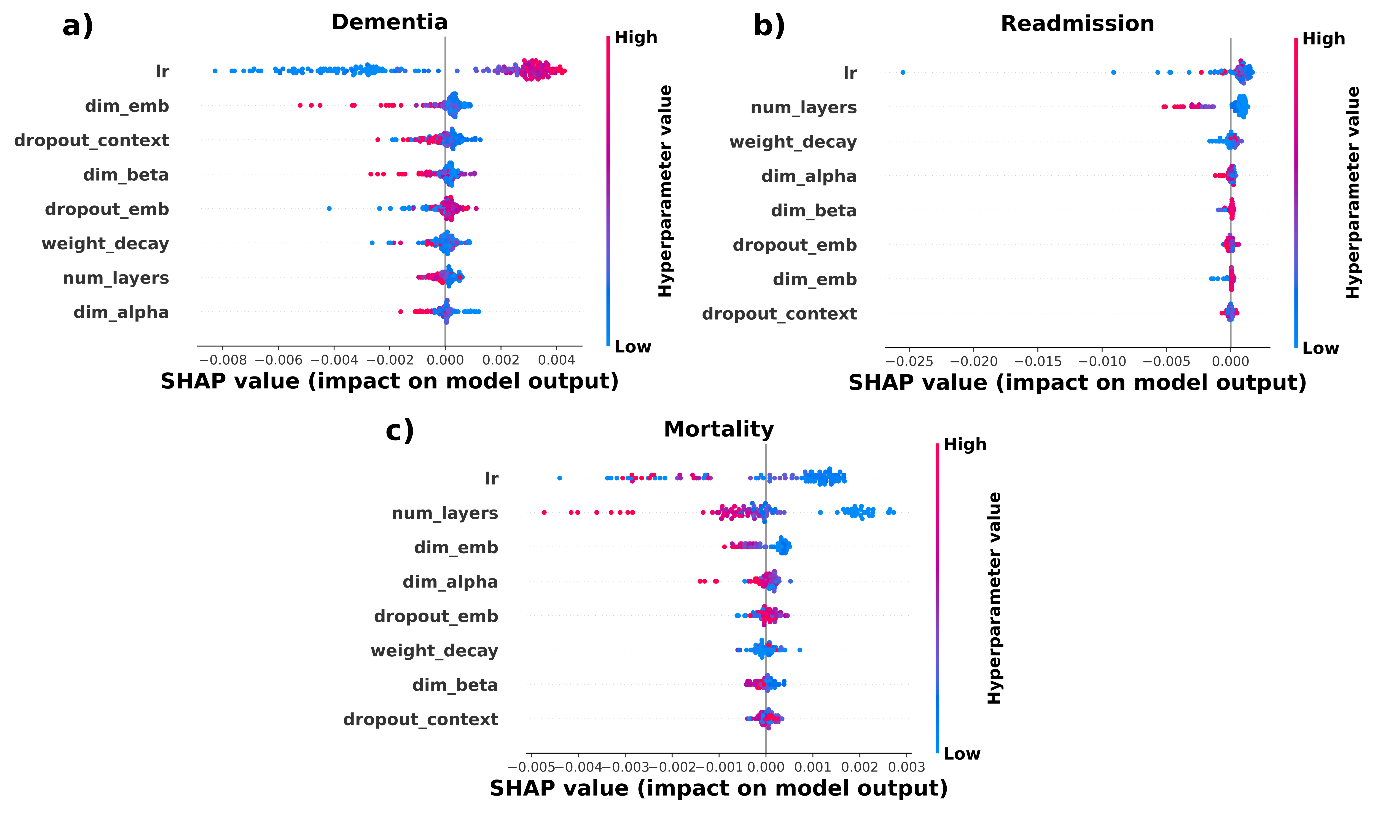


**Supplementary Figure 2:** Feature importances for RETAIN hyperparameters for a) Dementia, b) Readmission and c) Mortality prediction problems. On the y-axis are the hyperparameters while in the x-axis is the impact of them on the model output (validation AUC). Red means higher values of the hyperparameter while blue means lower. lr: learning rate, dim_emb: embedding dimension


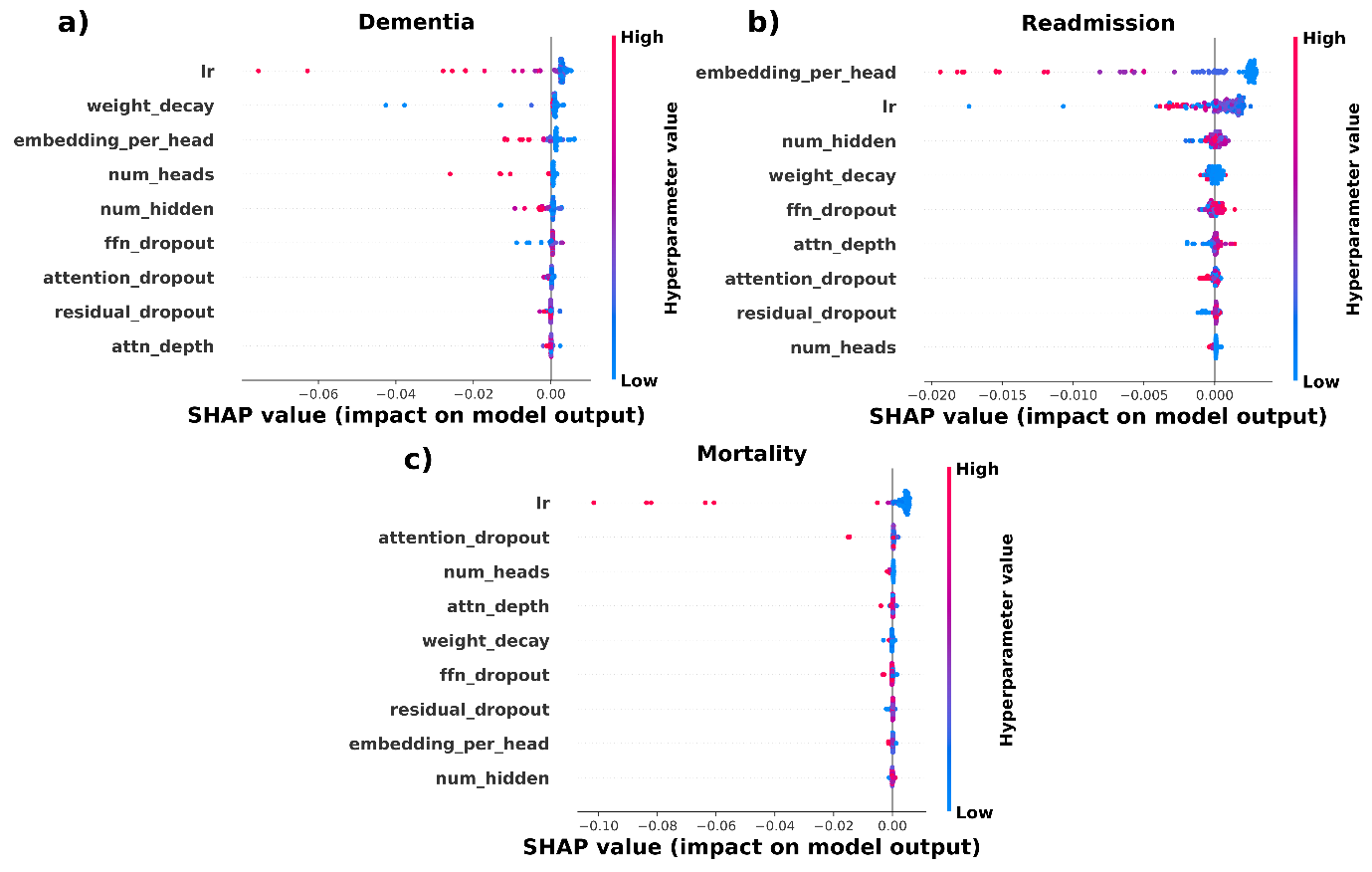


**Supplementary Figure 3:** Feature importances for the Transformer model for a) Dementia, b) Readmission and c) Mortality prediction problems. On the y-axis are the hyperparameters while in the x-axis is the impact of them on the model output (validation AUC). Red means higher values of the hyperparameter while blue means lower. lr: learning rate, ffn_dropout: Feedforward layer dropout


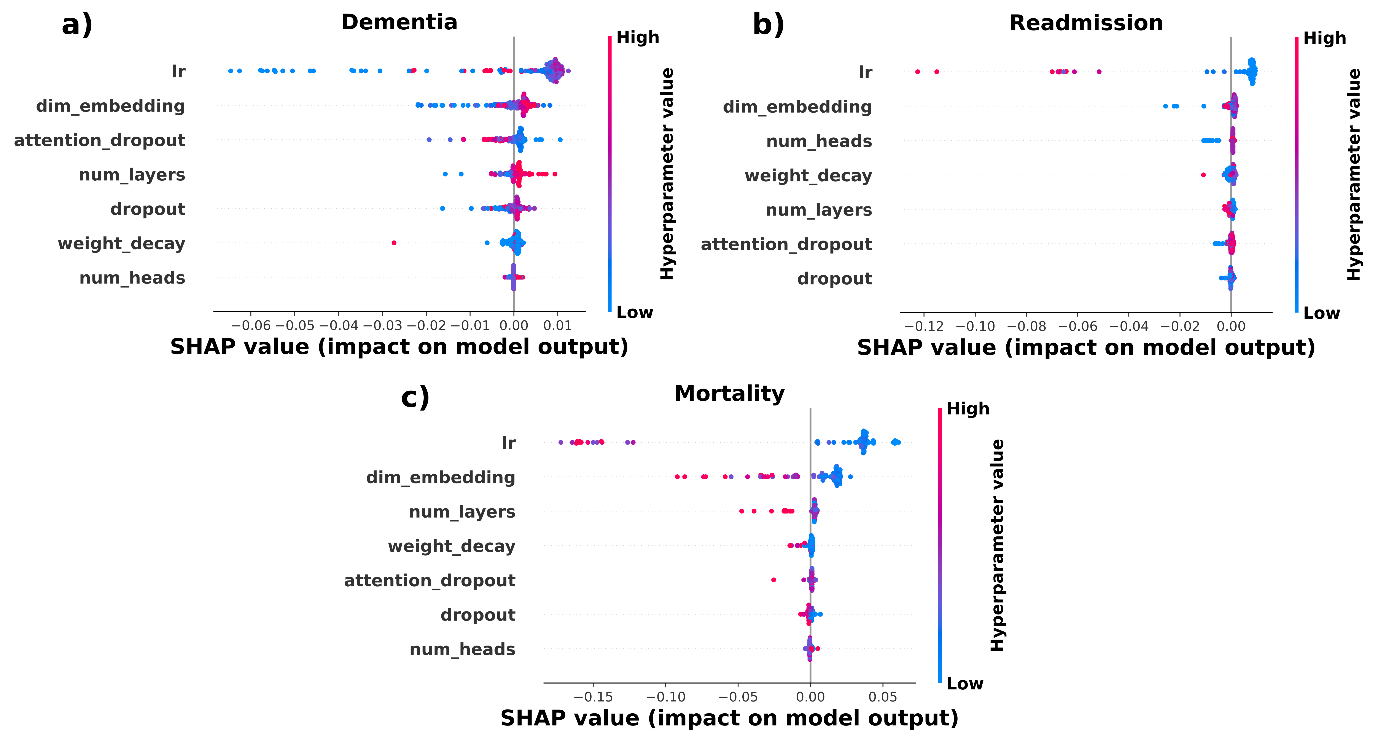


**Supplementary Figure 4:** Feature importances for the GNN model for a) Dementia, b) Readmission and c) Mortality prediction problems. On the y-axis are the hyperparameters while in the x-axis is the impact of them on the model output (validation AUC). Red means higher values of the hyperparameter while blue means lower. lr: learning rate
